# Artificial intelligence and digital medicine for integrated home care services in Italy: Opportunities and limits

**DOI:** 10.3389/fpubh.2022.1095001

**Published:** 2023-01-05

**Authors:** Mariano Cingolani, Roberto Scendoni, Piergiorgio Fedeli, Fabio Cembrani

**Affiliations:** ^1^Department of Law, Institute of Legal Medicine, University of Macerata, Macerata, Italy; ^2^School of Law, Legal Medicine, University of Camerino, Camerino, Italy; ^3^Operative Unit of Legal Medicine, Provincial Authority for Health Services of Trento, Trento, Italy

**Keywords:** artificial intelligence, home care service, telemedicine, telerehabilitation, limits

## Abstract

Home healthcare in the Italian health system has proven to be an essential factor in adequately responding to the health needs of an increasingly aging population. The opportunities offered by digitization and new technologies, such as artificial intelligence (AI) and robotics, are a lever for making home care services more effective and efficient on the one hand, and on the other for improving remote patient monitoring. Telemedicine devices have enormous potential for telemonitoring and telerehabilitation of patients suffering from chronic disabling diseases; in particular, AI systems can now provide very useful managerial and decision-making support in numerous clinical areas. AI combined with digitalization, could also allow for the remote monitoring of patients' health conditions. In this paper authors describe some digital and healthcare tools or system of AI, such as the Connected Care model, the Home Care Premium (HCP) project, The Resilia App and some professional service robotics. In this context, to optimize potential and concrete healthcare improvements, some limits need to be overcome: gaps in health information systems and digital tools at all levels of the Italian National Health Service, the slow dissemination of the computerized medical record, issues of digital literacy, the high cost of devices, the poor protection of data privacy. The danger of over-reliance on such systems should also be examined. Therefore the legal systems of the various countries, including Italy, should indicate clear decision-making paths for the patient.

## Introduction

Integrated Home Care (IHC) is a service available throughout Italy (1), structured to guarantee health and social assistance to elderly or sick citizens of all ages and social conditions, who are placed in family contexts suitable for providing the care they need at home.

Home care for elderly and/or non-self-sufficient patients is a priority for the Italian National Health Service (NHS), mainly for two reasons. On the one hand, it responds to the need to cope with the growing demands for health services related to the aging of the population, by providing patients with medical, rehabilitation and nursing services at home, thus significantly improving their quality of life in their family environment (13). On the other hand, it relieves the burden on hospitals, reducing emergency room admissions and inappropriate hospitalizations, which cuts costs for the National Health System (14).

Healthcare administered by IHC services at the patient's home is multidisciplinary. After a multi-professional team (Multidimensional Evaluation Unit) has completed an assessment, an Individual Care Plan (ICP) is drafted, and these steps call for the assistance of:

- Doctors (GPs or specialists)
- Nurses
- Physiotherapists or other rehabilitation professionals
- Psychologists
- Healthcare assistants.

Indeed, responding to the complex health and social needs of a typical multipathological patient requires the involvement of multiple medical professionals. The most frequently expressed need is to have a home nurse where necessary.

Although health policies in Italy have provided for home care for decades, IHC continues to play a marginal role and to be greatly insufficient compared to the real needs of the population. In fact, in 2021 only about 3% of Italians aged 65 and over were assisted at home, in view of a total of 3 million people suffering from multiple chronic conditions (MCC) and disabilities required continuous care (2). This is a very small percentage compared with other European countries, especially those of Northern Europe. Implementing the diffusion of new IHC systems remains a fundamental goal of the Italian NHS. The interest in the technological frontier of AI is consolidating more and more internationally and public health is an area that arouses great interest, especially where it is decided to intervene to improve the duration and quality of life (15). In addition, the importance of telemonitoring the patient both in healthcare facilities and at home is an even more pressing priority during the last years of the pandemic (16).

## Digital healthcare tools

The aging of the population (on 31 December 2021 Italian inhabitants aged 65 and over represented 23.2% of the total population, those up to 14 years of age 13% and those in the 15–64 age group 63.8%, while the average age approached 46 years) (3), an increase in chronic diseases and limited economic and human resources have put pressure on the health system, necessitating change in the form of better health services for patients, more efficient assistance from professionals and rationalization of economic resources.

**Figure 1:**
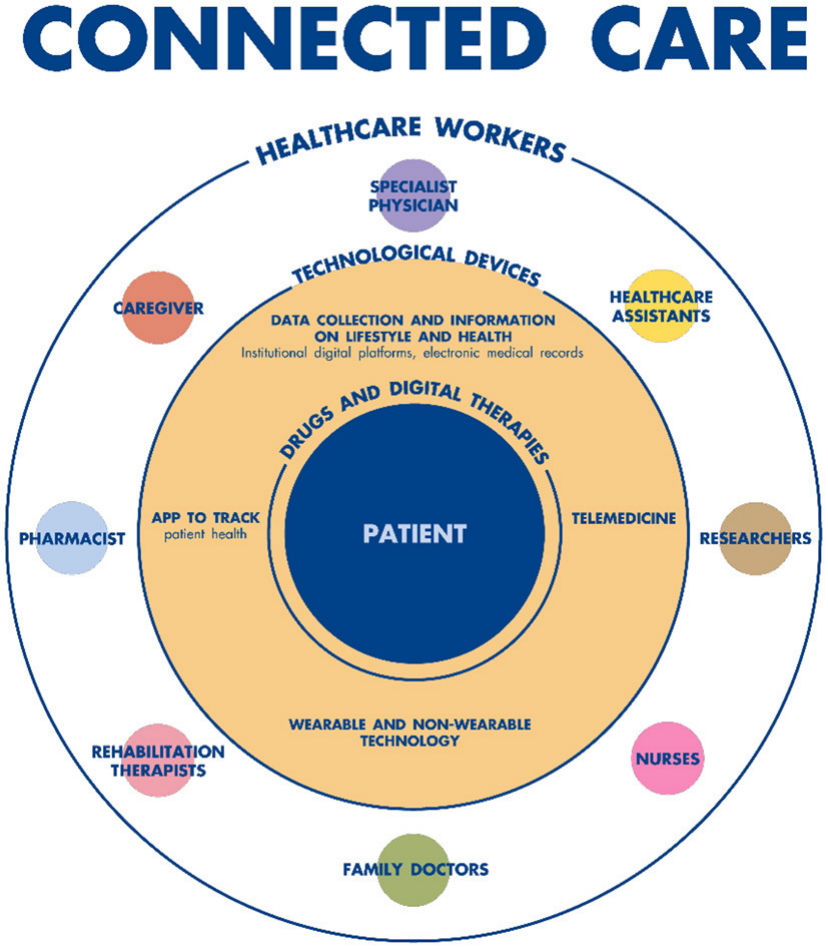
Connected care operating model.

Digitization is one of the major drivers of innovation and may be the solution to meeting the challenge of sustainability in the healthcare sector (17). Connected Care (4) (Figure 1) is gradually acquiring a strategic role in Italian digital healthcare, which puts the citizen-patient at the center of the system by creating organizational models that favor integrated care, between hospital and territory, to foster patient empowerment (18). This system includes new organizational models and technological solutions, in order to enable the sharing of patients' clinical information among all the actors involved in the treatment process (hospital doctors and nurses, local and home health workers, patients, insurers, institutional representatives, etc.); it is intended to be an operating system on which almost all institutions converge at central (Ministry of Health, MEF, Agid, etc.) and local (Regions and Health Authorities) levels to meet new health needs and maintain the balance of the health system.

The objective of Connected Care is to put the citizen-patient at the center of the system and to create organizational models that favor integrated care between hospital and territory, to enable patients to become more empowered. New technologies are fundamental in that they offer the means to enhance services and relationships between patients and health workers, from accessing health data and using services, to monitoring the clinical course and the overall evolving (or stabilized) state of health. Furthermore, based on the analysis of statistical models, such technologies can help to identify preventive behaviors.

The Italian NHS is therefore moving toward a new model of integrated and connected healthcare, with a clear path mapped out for its implementation and dissemination. To achieve this, it is necessary to combine different care models, intrinsically integrating the support of technology to ensure continuity of care for all patients, especially those who are chronically ill.

In this era of digitization, there are many ways to organize home healthcare. A valuable tool that has been introduced in Italy is the Resilia app (5), a mobile phone application which allows users to quickly find nursing care or other health professionals, such as social health workers and physiotherapists.

The Resilia app is very easy to download from the Google Store or Apple Store, depending on the operating system. It is simple to use and guarantees absolute security and privacy for all users. Once a person has registered as a user, they can use either their Google or Facebook account to complete the activation with a few pieces of information, and the service will be immediately active. Navigating through the menu, it is possible to select a geographical area and type of homecare support (e.g., nursing) to find out which services are available and how much they cost. The idea of developing an app that would connect patients to healthcare professionals and to all psycho-physical wellness operators stemmed from the difficulties encountered in accessing the services of a trusted professional (19, 20).

## Potentiality and future perspectives of AI in the healthcare system

Evolving technologies, such as AI, have the potential to help healthcare systems around the world respond to the major challenges they face, such as an aging population and the rise of chronic diseases, while increasing their sustainability. Systems equipped with automation mechanisms can help doctors improve diagnoses, perform surgical procedures, predict the spread of diseases and personalize treatments, thereby making a significant contribution to precision medicine–an emerging approach to the treatment and prevention of diseases that takes into account the individual variability in genes, environment and lifestyle to develop “tailor-made” treatments. Thanks to cognitive “supercomputers” capable of analyzing huge amounts of data, it is possible to make early diagnoses, as well as identify life-saving therapies much faster than traditional methods (21). Artificial intelligence, combined with digitalization, could also allow for the remote monitoring of patients' health conditions, thus potentially expanding the home care system (22). Similarly, the implementation of digital technologies in patients' homes could concretely and increasingly transform and improve healthcare in general and the home care system in particular. Intelligent machines are being developed to monitor correct adherence to medication and, more generally, the state of health of the elderly. Not only that, they could assist the patient in rehabilitation or in simple daily movements, such as getting out of bed, or alerting medical and nursing staff in case of need (23). From this point of view, AI and robotics truly have the potential to significantly improve the lives of millions of elderly and non-self-sufficient patients (24).

In this field, professional service robotics has been developed with very sophisticated humanoid machines, capable of providing direct assistance to people with dementia, thus replacing or complementing the assistance provided by caregivers. Fraunhofer IPA's Care-O-bot (now in its fourth generation, Care-O-bot 4) is a cross-platform interactive mobile robot, successfully tested to help with memory deficits and to support seniors in carrying out daily tasks (25). Another humanoid robot, Abel, resembles a young boy and is able to understand human emotions, make decisions and converse (26). A Japanese-designed humanoid robot, known as Pepper, has similar functions; it is capable of recognizing human faces and basic emotions thanks to emotion engine software (27). With machine learning programming, Pepper interacts and learns to become a social robot-caregiver that continuously improves its empathic skills and bidirectional interactions, learning to read and interpret emotions and then to react appropriately, thanks to the system of emotion classification.

AI tools represent an emerging field of application in healthcare, especially with regard to frail people, the elderly and those with chronic diseases who may benefit from remote patient monitoring, the prevention of critical situations and assistance with daily activities (28–30). Below is a list of what can be considered operational proposals, planned or partly prepared by the Italian health system:

- AI tools should be promoted in combination with telemedicine, to make it possible to expand the home care system (6, 7, 31);
- A digital hospital-doctor-territory network should be activated to monitor patients suffering from chronic diseases and to promote prevention through digital medicine systems;
- Guidelines should be given for the correct treatment of complicated situations (e.g., bladder catheters, ostomies, PEGs, difficult wounds) through all means available to the scientific community, such as newsletters, scientific journals, meetings and face-to-face and online events;
- Training courses should be planned for caregivers, including tutorial videos, with the aim of managing and empowering the patient at home as much as possible;
- Big data in healthcare systems and deep learning techniques should be used for the purpose of effective predictive and preventive medicine, thus acting long before the onset of symptoms for chronic and worsening diseases. In fact, instant access to the entire set of data would make it possible to predict the evolution of the clinical picture through decision support algorithms that could make the entire diagnostic-therapeutic-care process more efficient;
- A diagnostic-assistance model should be developed, based on the creation of a personalized electronic health record, capable of responding to requests for increasingly effective, efficient and quality diagnostic, prognostic and treatment services for the patient. Two ministerial decrees (8) were recently published to give a certain basis to the application of the Electronic Health Record (EHR), a tool of fundamental importance for digital healthcare. All of this can be achieved by emphasizing the constructivist nature of the process, aimed at bringing significant advantages to all stakeholders interested in the individual's care and assistance pathway. This would also lead to savings and better management of individual cases (32).

An interesting attempt to organize assistance for the non-self-sufficient person at home is the Home Care Premium (HCP) project, managed by the National Social Security Institute (INPS) and aimed at public employees and their family members who have a disability or are non-self-sufficient (33); the so-called “prevailing services” consist of monthly financial contributions to help reimburse expenses incurred for the remuneration of caregivers who support daily life activities. Then there are the “supplementary services” to support the daily care pathway: they consist of professional services at home and out of the home to enhance abilities, prevent or slow down the degeneration of the level of non-self-sufficiency and support ancillary assistance services, adapted to the level of non-self-sufficiency and socio-assistance needs (9). The healthcare worker is entrusted with the task of defining the potentially usable resources for each activity of daily life (ADL), in relation to the care that the person needs. In addition to a family assistant, home services of a medical or non-medical nature can be provided, as well as the possible installation of equipment at home (various aids) or home automation technological tools for mobility and autonomy to better manage the home environment and communications.

The Italian state is moving in this direction, and the welfare reform envisaged by the National Recovery and Resilience Plan (PNRR) has become law (10). Mission 6 of the PNRR (11), which stemmed from the need to bridge territorial disparities and offer greater integration between health services in different care settings, is dedicated to health and divided into two components:

- Component 1: Proximity networks, intermediate structures and telemedicine for territorial healthcare;
- Component 2: Innovation, research and digitalization of the national health service.

The possibility of accessing (freely and without discrimination) the new options offered by the extraordinary developments of intelligent assisted technologies (IAT) is an extremely important issue for the protection and promotion of human rights (34). As set out in the delegated law, it is necessary to precisely define the concept of disability. The Italian Society of Forensic Medicine, in a recent position paper, stated: “*A person with a disability is anyone who has a stabilized or progressive impairment of the integrated physical, psychic, intellectual and sensorial functions, or who suffers from a morbid process, even of a short duration, which seriously affects the integrity and efficiency of the person, causing the loss of personal autonomy. The impairment, interacting with barriers of a different nature, may hinder the full and effective social-relational-working participation of the person by inducing inequality and/or direct or indirect discrimination*” (12).

What further opportunities can we find in these new AI systems? (35).

a) The introduction of AI-enabled technologies, will allow family members and the care team to increase the level of communication with each other regarding better care for their loved ones aged.
b) Some robots can also remind seniors if social events occur in the neighborhood, encouraging them to go out and socialize.
c) Installing AI-powered sensors at home can also identify if a senior has fallen or has encountered an accident.
d) Many of the AI apps available on smartphones today could monitor health data, such as the elderly's daily activities, diet, and even lifestyle, in a less intrusive way.

## Limits and criticalities

The question arises as to whether the extraordinary options offered by AI open up avenues for those in real need, or whether the ways in which the technological tools are distributed are affected by prejudice that differentiates and hierarchies people, without agreed rules–not bound by any scruples. Intelligent assistive technologies (IAT) have made great strides; until recently, the areas of rehabilitation/inclusive intervention were usually limited to incontinence aids, active mobilization systems (wheelchairs and lifts), positioning aids (self-elevating armchairs) and aids for the prevention of pressure sores (water mattresses and pillows or the more innovative air mattresses with interchangeable elements). However, this does not rule out the possibility of new critical issues related to: the safety and reproducibility of the software; ethical-legal problems linked to the protection of the privacy of the data subject; and, above all, civil liability for any damage caused by the manufacturer and/or programmer (36, 37): if an AI system fails to deliver a rehabilitation treatment, or worse still, harms the patient, who is liable? The developer? The producer? The distributor? The programmer? The healthcare professional who made the decision to use it? The patient who uses it? The family member who puts it into operation? These issues are known in other fields of application of AI and have motivated legal positions on both national and international levels (38). In this regard, the legal systems of various countries, including Italy, should probably outline clear decision-making paths that are guaranteed for the patient.

The danger of over-reliance and excessive dependence on such systems should also be highlighted and investigated, for this could lead to serious effects such as the deskilling and desensitization of healthcare personnel. In other words, some decision-making processes could be influenced by new technologies, undermining the essential doctor-patient relationship (39).

Another matter of concern is the protection of privacy and security (40). In this context, there is unanimous agreement that the implementation of AI must be accompanied by careful reflection on the part of the legislator to ensure that the rights of citizens and patients are truly protected. For example, there is the question of consent to the processing of personal health data by artificial intelligence systems.

Finally, there are further limitations and criticalities to point out (41):

- While AI technologies can allow a lengthening of the patient's life, on the other hand it should avoid compromising the quality of health and social care that people who get older and older receive;
- The big data used may be unrepresentative of older people or distorted by past age stereotypes, prejudices or discrimination;
- There may be a risk of a reduction in intergenerational contact.

## Conclusions

The massive deployment of intelligent tools for physical, cognitive and behavioral assistance, as well as for the monitoring and provision of subsidized healthcare, will necessarily respond to social needs by allowing the elderly to continue living at home, while maintaining a residual degree of independence even when they live in sheltered care accommodation. There is a triple-win effect because these technologies are able to:

- Delay or obviate the need for institutional care, thus reducing the healthcare costs associated with long-term care and institutionalization;
- Mitigate the burden of care that often weighs on the family or other informal assistants;
- Improve the quality of life of the elderly who are not longer self-sufficient by supporting their independence, autonomy, social interaction and their right to age without being institutionalized (42).

Researchers, politicians and health professionals around the world have high hopes that assistive technologies can support the elderly, even in the face of questions that remain unanswered (43). Many devices are available for free on the market and are used widely even if research is ongoing to improve their effectiveness. However, the use of new enabling technologies and the consequent need for a reorganization of health services impose the urgency of applying systematic frameworks to increase the quality and safety of health services (44).

## Data availability statement

The original contributions presented in the study are included in the article/supplementary material, further inquiries can be directed to the corresponding author.

## Author contributions

MC and RS drafted the document (both as first authors) and acquired the information. PF made a substantial contribution to the conception of the work. FC analyzed the regulatory information and reviewed it critically. All authors contributed to revising the manuscript and approved the submitted version.
